# Microsaccades in Applied Environments: Real-World Applications of Fixational Eye Movement Measurements

**DOI:** 10.16910/jemr.12.6.15

**Published:** 2020-05-15

**Authors:** Robert G. Alexander, Stephen L. Macknik, Susana Martinez-Conde

**Affiliations:** SUNY Downstate Medical Center, Brooklyn, NY, USA

**Keywords:** microsaccades, drift, fixational eye movements, usability, real world applications, real world scenarios, occupational health, occupational safety, fatigue

## Abstract

Across a wide variety of research environments, the recording of microsaccades and other fixational eye movements has provided insight and solutions into practical problems. Here we review the literature on fixational eye movements—especially microsaccades—in applied and ecologically-valid scenarios. Recent technical advances allow noninvasive fixational eye movement recordings in real-world contexts, while observers perform a variety of tasks. Thus, fixational eye movement measures have been obtained in a host of real-world scenarios, such as in connection with driver fatigue, vestibular sensory deprivation in astronauts, and elite athletic training, among others. Here we present the state of the art in the practical applications of fixational eye movement research, examine its potential future uses, and discuss the benefits of including microsaccade measures in existing eye movement detection technologies. Current evidence supports the inclusion of fixational eye movement measures in real-world contexts, as part of the development of new or improved oculomotor assessment tools. The real-world applications of fixational eye movement measurements will only grow larger and wider as affordable high-speed and high-spatial resolution eye trackers become increasingly prevalent.

## Introduction

To date, most studies of fixational eye movements—FEMs, the small movements we make while we attempt to fixate our gaze on a target—have been conducted in laboratory settings. Though invaluable for their controlled and reliable conclusions about well-defined contexts, such restricted scenarios may provide limited insight into the roles and applications of FEMS in real-world conditions.

FEMs consist of three main kinds of movements: microsaccades, drift, and tremor. Of these, microsaccades have been best studied, largely due to their comparatively larger sizes and speeds, which make them easier to detect and characterize than drift and tremor —especially with noninvasive commercial video trackers. 

Thus, changes in microsaccadic dynamics have been linked to changes in perception (1, 2, 3, 4) and attention (5, 6, 7, 8, 9, 10), as well as to neurologic and ophthalmic conditions (11, 12, 13, 14, 15, 16, 17). 

Extending and expanding this research into ecologically-valid contexts has been particularly important because debates about FEMs have often been ecological in nature: For example, after early studies demonstrated the existence of microsaccades, some researchers questioned whether these movements were a laboratory artifact without any real-world function (e.g., 18, 19). We now know that microsaccades are not merely an artificial response to unnatural experimental conditions, and we understand some of the contributions of microsaccades to perception and cognition (20, 21, 22, 23). For example, microsaccade rates and amplitudes can indicate working memory load (24, 25), task complexity (6), or the informativeness of different image regions (26, 27). 

Accordingly, a growing body of research has started to highlight multiple real-world implications of microsaccades—and to a lesser extent drift—, from monitoring of fatigue to the prevention of occupational health and safety hazards, to the identification of differences in perceptual judgments by novice and elite athletes. Thus, FEM measures in applied domains provide researchers with valuable information about the dynamics of cognitive and perceptual performance within those domains, while helping to clarify the real-world functions of FEMs. Here, we offer a comprehensive review of the literature on applications of FEMs to ecologically-valid scenarios and real-world contexts. Due to the difficulty of measuring tremor, even inside the lab, and the resulting scarcity of studies including tremor measurements, this review focuses primarily on microsaccades, and secondarily on drift. Table 1 lists the microsaccade detection criteria and the eye tracking methodologies used in the studies we examine here.

**Table 1:** Comparison of eye-tracking methods and FEM detection in applied contexts.

Applied context	Eye-tracking system	Sampling rate	Head position restraints	FEMs measured	Microsaccade detection algorithm	Binocularity requirement for microsaccade detection	Maximum microsaccade amplitude	Reference
Air traffic control	EyeLink 1000 (SR Research)	500 Hz	Forehead and chin rest	Drift and microsaccades	Engbert and Kliegl (5) algorithm	Yes	<1°	Di Stasi, McCamy (28)
Athletic performance (table tennis)	Head-mounted EyeLink II (SR Research)	500 Hz	Chin rest	Microsaccades	Engbert and Kliegl (5) algorithm	Yes	<1°	Piras et al. (2015)
	Head-mounted EyeLink II (SR Research)	500 Hz	Chin rest	Microsaccades	Unsupervised clustering method (29)	Yes	<1°	Piras, Raffi (30)
Aviation	EyeLink 1000 (SR Research)	500 Hz	Forehead and chin rest	Drift; Microsaccades not explicitly tested, but small saccades included in analysis	Engbert and Kliegl (5) algorithm	Yes	N/A	Di Stasi, Cabestrero (31)
	Head-mounted Dikablis Professional eye-tracker (Ergoneers, Inc.)	60 Hz	None	Microsaccades	D-Lab 3 software	No	<1°	Thropp and Buza (32)
Driving	Head-mounted SMI X-HED monocular eye-tracker (SensoMotoric Instruments)	200 Hz	None	Microsaccades	Velocity-threshold identification from SMI BeGaze 2 software	No; recorded monocularly	<1°	Benedetto, Pedrotti (33)
	Eyelink 1000 (SR Research)	500 Hz	None	Microsaccades	Engbert and Kliegl (5) algorithm	No; recorded monocularly	<1°	Di Stasi, McCamy (34)
	EyeSeeCam	~220 Hz	None	Microsaccades	Horizontal velocity and acceleration thresholds set separately for each subject	No	<1°	Miki and Hirata (35)
	Head-mounted JAZZ-novo (Ober Consulting, Poznan, Poland)	1 KHz	None	FEMs not explicitly tested, but small saccades included in analysis	Engbert and Kliegl (5) algorithm	Yes	N/A	Morales et al. (36)
High-acuity tasks (e.g., sewing and shooting)	Modified photocell system (37)	Not specified	Bite bar	Microsaccades	Not specified	Not specified	<15’ arc	Bridgeman and Palca (38)
	DPI eye tracker	1 kHz	Bite bar and head rest	Drift and microsaccades	Speed threshold (>3°/s) and amplitude threshold (>1’)	No; recorded monocularly	<20’ arc	Ko, Poletti (39)
	Head-mounted EyeLink II (SR Research)	500 Hz	Chin rest	Microsaccades	Engbert and Kliegl (5) algorithm	No; combined both monocular and binocular microsaccades	Not specified	Valsecchi and Gegenfurtner (40)
	Search coil	Not specified	Forehead, temple, and chin rest	Drift and microsaccades	Paper polygraph records measured with a magnifier and a scale	Not specified	<10’ arc	Winterson and Collewijn (41)
Spaceflight	EyeLink video oculography system (SensoMotoric Instruments)	≤ 240 Hz	Head fixed to a tilt chair	Microsaccades not explicitly tested, but small saccades included in analysis	Not specified	Not specified	N/A	Reschke, Somers (42)
	Electro-oculography	Not specified	Head fixed in a straight position using a collar and fixed to the monitor	Drift and microsaccades	Custom software	Not specified	Not specified	Kornilova (43)
	Electro-oculography, the VNG Ulmer videooculography system (Synapsys and Heinemann Medizintechnik), and a Visio 5.0 mask with a video camera	Not specified	Head fixed in a straight position using a collar and fixed to the monitor	Drift and microsaccades	Custom software	Not specified	Not specified	Kornilova, Alekhina (44)
	Electro-oculography	Not specified	Head fixed in a straight position using a collar; some data recorded with the collar fixed to the monitor	Drift and microsaccades	Not specified	Not specified	Not specified	Kornilova and Kozlovskaya (45)
	Head-mounted Eye Tracking Device (Chronos Vision) and electro-oculography	200 Hz	Head fixed in a straight position using a collar	Drift and microsaccades	Not specified	Not specified	Not specified	Kornilova, Naumov (46)
Magic performance	EyeLink 1000 (SR Research)	500 Hz	Chin rest	Microsaccades	Engbert and Kliegl (5) algorithm	Yes	<1.5°	Barnhart, Costela (47)
Viewing of video or other dynamic stimuli	Generation 6 Dual Purkinje Image Eyetracker (Fourward Technologies)	488 Hz	Bite bar and head rest	Drift	Estimated from the signals given by both head and eye coils	No	<30 arcmin	Roberts, Wallis (48)

### Microsaccades and drift in ecologically-valid contexts

Whereas the great majority of early studies relied on simple, artificial stimuli (but see Winterson and Collewijn (41)) and highly restrictive setups (including, for example, the use of bite-bars), FEMs have now been measured in a wide array of conditions with varying degrees of real-world validity: Beyond the classic fixation task, microsaccades have been shown to occur during the fixation periods that ensue during free-viewing, visual exploration, and visual search tasks (12, 26, 27, 49, 50). Moreover, FEMs have been quantified with head-unrestrained fixation (1, 51), with dynamic artificial stimuli (27, 52), during the observation of art (3), and during free-viewing of dynamic video (30, 48). Although some FEM parameters change with experimental context (for example, variations in task difficulty can produce changing microsaccade rates (6, 27)) some key characteristics of FEMs remain unchanged across varying research scenarios, as discussed below.

Microsaccades follow the saccadic “main sequence” relationship between amplitude and velocity, not only during prolonged fixation, but also in the fixation periods that occur between saccades during free-viewing and visual search (10, 20, 27, 53). Otero-Millan, Macknik (49) extended these findings into ecological viewing conditions, in which participants gazed at a small dot or freely viewed natural scenes of varying sizes. Microsaccades followed the saccadic main sequence even in conditions where participants were able to move their eyes and heads naturally, while exploring a display that encompassed the virtual entirety of the visual field (up to 160 degrees horizontally).

Another area of interest in microsaccade research, which has started to extend to more realistic experimental contexts, is the relationship between microsaccadic features and covert attention, the shifting of attention without a corresponding shift in gaze. After some early debate, there is overall consensus in the field that attention and microsaccades are intimately related, with biases in microsaccade direction pointed to the spatial location of covert attention in the visual field (7, 8, 20). 

Barnhart et al observed biases in microsaccade direction in the ecologically-valid context of attentional misdirection during the performance of magic (Barnhart, Costela, Martinez-Conde & Goldinger, 2019). Participants viewed a magic trick where a coin vanished from beneath a napkin and reappeared under a different napkin. Microsaccade direction served as an index of covert attention, helping researchers determine how participants divided their attention across the two napkin locations, while being fooled by the magician’s misdirection. 

Microsaccade direction has also been linked to social orienting in both humans and rhesus macaques. Guerin-Dugue, Roy (54) found that pictures of rhesus macaques looking in a certain direction prompted both humans and rhesus macaques to covertly orient their attention in that same direction. Though the direction of the macaque’s gaze in the images was task-irrelevant, it did bias microsaccade direction in a congruent manner. The authors concluded that such social orienting took place reflexively, in response to the gaze directions portrayed in the images.

 Several recent studies have moreover used microsaccade direction to assess differences between the attentional focus of novice versus elite athletes (further discussed later in this review; (30, 55)). 

Attempts have also been made to quantify drift parameters in ecologically valid conditions. Recent work has replicated with dynamic stimuli the previous finding that drift can be successfully modelled as a self-avoiding random walk (56, 57). To achieve this, Roberts, Wallis (48) asked participants to view the Alfred Hitchcock film “Rope”, selected because the film has only ten director’s cuts throughout the entire 77 min of continuous video (thus providing a naturalistic stimulus), while tracking their eye movements. These conditions allowed the researchers to observe short-term “superdiffusive processes” (where the gaze wanders faster than expected, which may help refresh retinal images (58). Superdiffusive processes during drift had been demonstrated previously with static images, but not during the viewing of dynamic videos.

As the use of natural, ecologically-valid stimuli grows, the usefulness of FEM measures in a diversity of research scenarios is likely to become increasingly apparent. One consideration with many potential practical applications is that, whereas microsaccade magnitudes are relevant for close inspection of small objects or object features at short viewing distances, the same slight changes in angular eye rotation may shift an observer’s gaze between large objects at longer viewing distances Poletti and Rucci (59).

### Microsaccades and driving

The field of automotive design holds great promise for the application of FEM measures: eyesight is the primary source of information during driving (60), and eye movement biomarkers may help prevent accidents by assessing fatigue, distraction, and other risk factors (61). 

Benedetto, Pedrotti (33) measured saccadic movements—including microsaccades—to assess the level of distraction drivers might experience on the road. Participants performed a divided attention task, changing lanes in a driving simulator while simultaneously locating targets in a visual search task displayed via an In-Vehicle Information System (IVIS). Microsaccade rates increased in connection with attentional shifts between the lane-change task on the simulator and the visual search task on the IVIS.

Miki and Hirata (35) tracked the eye movements of participants driving a real car on an open road, to determine whether microsaccades could co-occur with the vestibulo-ocular reflex (where movements of the head cause opposing movements of the eyes, so that gaze is maintained on a location). Though prior research had established microsaccade generation in head-unrestrained conditions (1, 51), this study set out to determine if microsaccades could be produced—or instead, were suppressed—while the head was unrestrained *and moving*. Participants wore a head-mounted EyeSeeCam eye tracker and followed a set driving route around the Chubu University Kasugai campus. The vestibulo-ocular reflex altered the gaze data during the drive, as the head moved by itself or as a result of changes in elevation of the road. Microsaccades were detected—though at much lower rates than in other studies—including during the compensatory slow-phase of the vestibulo-ocular reflex.

#### Microsaccades and driving fatigue:

Mental fatigue is a common cause of errors in critical situations such as while driving, flying a plane, or performing surgery. Multiple research studies have found saccadic and microsaccadic eye movement measures to be valid and objective indicators of fatigue in a variety of scenarios, both inside and outside the lab (61-66). 

**Figure 1.**
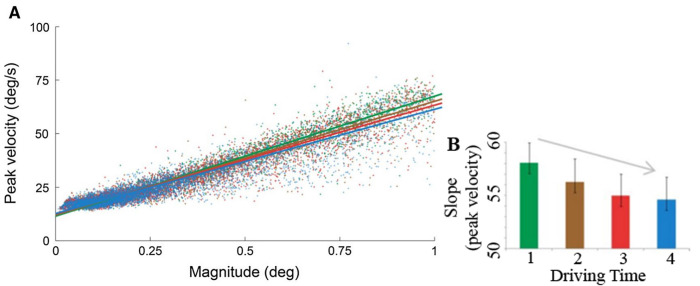
**A)** Microsaccadic main sequence for one participant as a function of four binned driving times (1: green, 2: brown, 3: red, 4: blue; 30 min durations per bin). **B)** Average microsaccadic peak velocity for all participants for each 30 minutes of driving time (shown in the same bin colors as A). The significant linear trend of microsaccadic velocity over time is indicated by the arrow. Error bars indicate SEM across participants (n=7). Modified from Di Stasi, McCamy (34).

Di Stasi, McCamy (28) first found microsaccadic dynamics of fatigue, and subsequent studies extended their findings into real-world scenarios. Di Stasi, McCamy (34) recorded eye movements during a 2-hour simulated driving task. They found that microsaccadic velocities decreased with time spent driving, presumably due to increasing mental fatigue—see Figure 1. In line with previous work (34, 61), Morales, Díaz-Piedra (36) also found that increasing driving times resulted in decreased (micro)saccadic velocities. Longer driving times were moreover accompanied by increased speeding (over the speed limit), along with changes in the power spectra of the beta electroencephalogram (EEG) band. These data indicate that (micro)saccades are a potential avenue to accurately measure fatigue during driving.

### Microsaccades and drift in aviation

As with driving, fatigue is a cause of accidents and errors in aviation, and studies of FEMs in aviation have focused on detecting changes in fatigue. Di Stasi, McCamy (67) determined that saccadic velocities could be used as a biomarker of fatigue and fitness-for-duty in aviators: Saccade velocities decreased after long >1 hour simulated flight missions (relative to shorter simulated flights). The eye movements measured included microsaccades, although significant differences due to fatigue were only apparent in the larger saccadic movements. 

#### Distinguishing fatigue from other factors

Acute hypoxia, defined as decreased oxygen in the body at the tissue level, is a serious hazard in aviation that can lead to visual dysfunction, rapid pulse, dyspnea, syncope, and mental disturbances including delirium and euphoria (68, 69, 70, 71). Subjective fatigue is a side-effect both of time on duty and of hypoxia. Thus, in some situations pilots may not be able to ascertain whether they are hypoxic or merely fatigued, until more serious side-effects develop (61, 71 , 72). Given the relationship between altered oculomotor dynamics and fatigue, Di Stasi, Cabestrero (31) wondered if eye movement measures could distinguish between short-term hypobaric hypoxia and fatigue—and thus provide a useful tool for their differential diagnosis. Spanish Air Force pilots and flight engineers conducted a guided-saccade task (following a jumping fixation target) before and after entering a hypoxic chamber. Critically, a control group performed the same guided-saccade task at equivalent times (~3 hours between measurements) but carried out their regular duties instead of being exposed to hypoxia. Thus, the researchers were able to dissociate the effects of hypoxia on FEM dynamics from those of time-on-duty, by comparing the measurements from both subject groups. Peak saccadic velocities decreased from the Pre-Test to the Post-Test session for both hypoxia and control groups, but this effect was not statistically significant after controlling for time-on-duty. This could help explain some disparate findings concerning the effects of hypoxia on oculomotor behavior: some researchers have likewise found no effect of hypoxia on saccadic velocity (73, 74), whereas others reported that hypoxia appeared to alter saccadic velocities (75, 76). Di Stasi et al. moreover found that drift velocities increased from the Pre-Test to the Post-Test session, and that such drift velocity increase was larger for the hypoxia group than for the control group. Their findings suggest that acute hypoxia diminishes eye stability independently of fatigue.

A more recent study has reexamined the relationship between microsaccadic velocity and hypoxia. By using a flight simulator housed within a hyperbaric chamber, Thropp and Buza (32) exposed pilots to decompression during simulated flight. The pilots were given periodic instructions to change heading, altitude, or other flight parameters. Microsaccade rate was found to increase with decreasing levels of blood oxygenation, suggesting that hypoxia may destabilize eye movements (consistent with the results of Di Stasi et al (2014)). However, the authors also found that saccadic velocity decreased with hypoxia, contrary to previous findings of either increases or no changes in saccadic velocity (31, 73, 74). The combined results of the above studies indicate that saccadic velocity differences between hypoxia studies are likely due to their differing task demands, rather than by hypoxia per se. Specifically, whereas the task in Thropp and Buza (32) was slow-paced, possibly leading to decreases in saccadic velocity, fast-paced tasks in prior studies may have caused increases in saccadic velocity.

### Microsaccades and drift in air traffic control

Air traffic control is another task in which fatigue can result in severe consequences (77). Decreased attentional levels associated with fatigue may cause operators to ignore or misread incoming information, thus compromising job performance and safety. Accordingly, Di Stasi, McCamy (28) asked whether FEM characteristics could serve to index fatigue while air control operators are on duty. Participants performed a simplified air traffic control task, intended to mimic the demanding visual search tasks performed by air traffic controllers. In different trials, subjects either fixated a central dot (which represented the airport) or freely moved their gaze over the entire display (the rest of which represented airspace). On each trial, participants reported whether or not there was a “conflict” (two triangles—representing aircraft—of the same color and on the same concentric circle). Ocular instability intensified with fatigue: as time-on-task increased, saccadic and microsaccadic velocity decreased, but drift velocity increased. Task difficulty (displays with 8 vs 16 planes) affected reaction time and accuracy, but not eye movement velocity. Thus, changes in FEM dynamics can signal mental fatigue irrespective of task difficulty.

### Microsaccades and drift in spaceflight

Observations of astronauts’ oculomotor behavior provide additional evidence of changes to FEMs in response to flight conditions. Yet, spaceflight differs from atmospheric flight in many ways, including a substantial difference in gravitational forces. On earth, the orientation of the head relative to gravity is constantly signaled by otolith organs, which respond to changes in linear acceleration (including the vertical acceleration due to gravity). Eye movements are modulated in response to changes in these signals as part of the vestibulo-ocular reflex, which maintains gaze stability when the head is moved. The low-gravity conditions present in spaceflight might therefore alter FEMs and decrease gaze stability.

Reschke, (2004) (42) presented a case study of a veteran astronaut (with ~140 days of experience on orbit), who showed frequent square wave jerks (SWJs; the most common kind of saccadic intrusion, consisting on (micro)saccades that take the gaze away from a foveated target, shortly followed by (micro)saccades that return the gaze to the target), without apparent perceptual deficits. Reschke et al. hypothesized that the astronaut’s extended exposure to low gravity conditions might have resulted in the elevated SWJ frequency. Though no prior measurements to the astronaut’s first exposure to low-gravity conditions were available for comparison, the authors noted that his already-high frequency of SWJs (pre-spaceflight) was further increased post-spaceflight, and remained elevated even a month afterwards. No changes in SWJ frequency were observed with respect to variations in tilt (with the astronaut seated in a tilt chair) relative to gravito-inertial forces, however.

Kornilova and Kozlovskaya (45) tested the effects of vestibular sensory deprivation (functional deafferentation of the otolith organs due to long exposures to microgravity) on the eye movements of 31 Russian cosmonauts. They tracked the cosmonauts’ eye movements (using an electrooculogram) before, during, and after spaceflight, on flights ranging from 7 to 438 days in space. Eye dynamics during spaceflight were assessed in freefall conditions, while the cosmonauts’ heads were fixed in place. The data, which were analyzed upon return to earth, revealed that prolonged periods spent in microgravity conditions caused a variety of oculomotor impairments. Gaze fixation alterations included increases in slow drift, in SWJs, and in spontaneous nystagmus. Smooth pursuit was also affected, and disappeared completely in 40% of the cosmonauts, who used a series of micro- and large saccades (instead of smooth pursuit) to track visual targets.

Kornilova (2004) again reported increases in drift in cosmonauts during spaceflight, as well as increases in nystagmus (both in spontaneous eye movements and while performing stepwise saccades to target points). In addition, the amplitudes of vertical stepwise saccades were reduced, relative to pre-flight baselines, so that 47% of the cosmonauts made additional (micro)saccades to reach the targets. Loss of smooth pursuit movements was also observed in some cosmonauts. Vestibular activation—via active head movements—resulted in improved smooth pursuit tracking, indicating that the oculomotor impairments observed were due to vestibular sensory deprivation from prolonged microgravity exposure (rather than from some other microgravity-related problem). 

In a follow-up study of cosmonauts during long spaceflights (126–195 days), Kornilova, Alekhina (44) again found similar oculomotor impairments, and noted a link between increased drift and nystagmus, and loss of smooth pursuit.

More recently, Kornilova and colleagues (46) found increased drift and SWJs two days after spaceflight. Oculomotor impairment was slightly more frequent in cosmonauts without prior spaceflight experience (33%) than in veteran cosmonauts (21%). In addition, novice cosmonauts displayed increased levels of drift and SWJs even nine days post-flight (by which point 100% of experienced cosmonauts had returned to baseline). Overall, reported symptoms of “space adaptation syndrome” (such as dizziness and spatial illusions) were strongly correlated with altered oculomotor dynamics, suggesting that gaze distortions in spaceflight could be manifestations of space adaptation syndrome and space motion sickness (in which space adaptation symptoms are serious enough to decrease the professional efficiency of astronauts).

Taken together, the studies above indicate that prolonged exposure to low gravity conditions causes gaze instability, with measurable alterations in drift, nystagmus, SWJs, (micro)saccades, and smooth pursuit movements, both during free viewing and in tasks that require the controlled allocation of gaze.

### Microsaccades and athletic performance

Over the past several decades, researchers have increasingly used eye tracking measurements to study cognitive and motor processes in athletes during sports performance (78). Few of these studies have conducted gaze analysis on the scale of FEMs, however, likely due to the difficulties inherent to measuring small eye motions during dynamic tasks. Sport-relevant stimuli necessarily consist of either live actors or dynamic videos, and players themselves move a great deal more than participants in standard psychophysical settings. Many sports cannot accommodate concurrent eye measurements, and even in situations when players are able to wear eye-tracking glasses, such glass-mounted eye-trackers are not best-suited for measuring FEMs. In early studies, conducted with larger eye movements, observers watched pre-recorded videos and made decisions about game situations (79). Similar scenarios and decision-making tasks have more recently been applied to the study of FEMs in sports, as discussed below. 

Piras et al. (2015) investigated microsaccade dynamics in table tennis players. A professional table tennis coach was filmed while swinging the racket and striking balls. Expert and novice players watched the coach hit the balls on video and tried to predict, after each hit, whether the balls would end on the left or right side of the table. While conducting this task, participants had to constrain their gaze to a constant fixation target in the dynamic scene: a red dot on the middle of the coach’s chest.

During the ‘post-bounce period’ (i.e. from when the ball bounced off the table until the coach’s racket hit the ball, presumed to be the time window with the greatest attentional load), both experts and novices made more frequent and larger microsaccades than in other time periods. In the case of incorrect predictions, the post-bounce microsaccade rates of experts and novices were comparable. In the case of correct predictions, the post-bounce microsaccade rates of experts were lower than those of novices. 

Whereas post-bounce microsaccade durations did not differ between experts and novices, experts made longer-duration microsaccades than novices during the ‘response period’ (i.e. from the coach's racket–ball contact to the observer's response), though only in the case of incorrect predictions. No differences in microsaccade rates were observed between experts and novices during the response period, however. 

Throughout the viewing task, observers were more likely to direct their microsaccades to the left than to the right of the fixation target—which was the side of the display that most often contained the ball or the racket. No relationship between microsaccade direction and response accuracy or expertise was found, for any task period. 

In a subsequent study by the same group, observers again viewed pre-recorded table tennis videos, but their fixations were unconstrained (30). That is, novices and expert players were allowed to move their eyes freely across the videos, instead of maintaining fixation on the coach’s chest. Eye movement analyses were limited to ‘interest areas’ surrounding the coach’s head, trunk, and hand/racket, as well as the ball. 

Experts made fewer and longer fixations than novices, perhaps due to having learned to “anchor” or peg their gaze on a given location for extended time periods in order to use *both *their foveal vision and parafoveal vision to sample the scene. Whereas experts had higher overall microsaccade rates than novices, both experts and novices made more microsaccades in more frequently fixated regions, consistent with prior reports of increased microsaccade production in consistently fixated natural scene regions (McCamy et al., 2014). 

During forehand strokes, the hand/racket area attracted higher microsaccade rates from experts, whereas the head area attracted higher microsaccade rates from novices. During backhand strokes, the trunk area attracted higher microsaccade rates from experts, whereas the head area again attracted higher microsaccade rates from novices. In addition, microsaccades were largest and fastest on the head and trunk regions, for both experts and novices. 

Microsaccade directions shifted between forehand and backhand strokes when both experts and novices fixated the ball, and also when experts (but not novices) fixated the hand/racket region. One possible explanation for this difference is that experts fixating the hand/racket region might allocate their covert attention to the opposite side of visual field (where the trunk/head are located), but that novices lack the skill to do so. 

Gonzalez, Causer (80) suggested that FEM measures may also provide important insights into the relationship between vision and complex movements underlying the “quiet eye” phenomenon, in which elite performers fixate or track critical spatial locations earlier and for longer times than other performers. Specifically, the “quiet eye” refers to the tendency for final fixation before a critical motor movement to be longer in duration for elite athletes than for near-elite or lower-skilled performers. Though the amplitude and frequency of FEMs during the quiet eye has yet to be explored, it could help researchers determine whether expert athletes’ enhanced attention to small spatial areas increases the precision of their visual targeting, or whether very precise fixations are not required to accomplish highly expert motions (if, for example, microsaccade rate during the quiet eye is higher for experts than for non-experts) (80).

###  Microsaccades during high-acuity tasks

One often-asked question in FEM research has been whether microsaccades help improve performance in tasks requiring high-acuity judgments, such as the threading of a needle. 

An early study by Winterson and Collewijn (41), aimed at determining whether microsaccades might be useful in *any* real-world tasks, measured the eye movements of participants while they aimed and fired an air rifle, or threaded a sewing needle. The experiments were designed to mimic the real-life scenarios as closely as possible, while providing objective measurements. Sewing task trials required participants to thread a copper wire into a needle. Trials started when the needle was illuminated, and ended as soon as the wire touched the needle (which closed an electrical circuit). Shooting task trials similarly began when the rifle was illuminated, and ended with the participant’s pulling of the trigger. As participants conducted these tasks, their microsaccade rates not only did not increase above baseline (2 microsaccades/sec, measured during stable fixation), but dropped to ~0.5/second at the end of both types of trials (needle-threading and rifle-aiming). This pattern suggested that microsaccades were not important to the performance of high-acuity tasks. 

In a follow-up “needle-threading” study, Bridgeman and Palca (38) asked participants to judge whether the tip of a horizontally oriented “thread” was moving towards a location above or below the tip of a stationary “needle,” without having them perform an actual motor task. Two tungsten microelectrodes—with tips smaller than the human eye can resolve—served as the “needle” and “thread.” Microsaccade rates again decreased over time, with the lowest frequencies occurring at the end of the trials, when participants made their judgements. Thus, Bridgeman and Palca arrived at the same conclusion as Winterson and Collewijn did previously: microsaccades did not contribute to high-acuity perception in real life contexts.

In contrast to these pioneering studies, later experiments showed that microsaccades can improve performance in active visual tasks, including high-acuity perceptual judgments. Ko, Poletti (39) reexamined the utility of microsaccades in the needle-threading task by having participants thread a virtual needle. They found that microsaccades precisely relocated the participant’s gaze back and forth between needle and thread throughout the trial. It was only towards the end of the trial, when participants had finished making adjustments to the relative positions of needle and thread, that microsaccade rates dropped.

**Figure 2.**
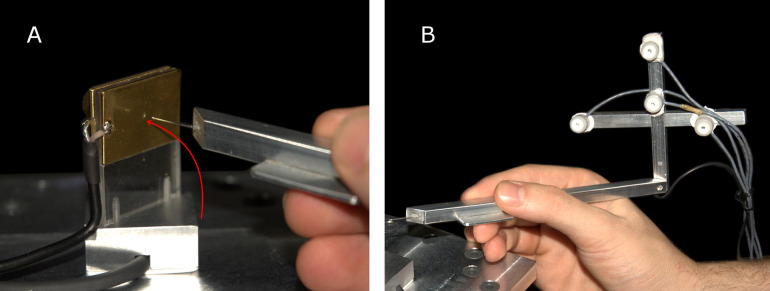
A) Participants in Valsecchi and Gegenfurtner (40) tried to touch a small (1 mm) hole in a metal plate with a thin needle. The instructed movement is illustrated in red. B) Four ultrasound-emitting markers enabled motion tracking of the needle tip’s spatial position. From Valsecchi and Gegenfurtner (40).

Valsecchi and Gegenfurtner (40) returned to a physical, real-world setup, to investigate the binocularity of microsaccades during high-precision tasks. In the main experiment, participants attempted to hit a target hole in a plate, using a hand-held needle—see **Figure 2**. Consistent with prior research (38, 39, 41), microsaccade rates decreased towards the end of the trial in the main experiment, when the tip of the needle approached the hole in the plate.

Contrary to the authors’ predictions, there was no coordination of microsaccadic version and vergence (i.e. microsaccadic displacements in depth and in the horizontal axis were not equal in amplitude). However, this coordination was found in a control experiment, designed for such specific purpose. Participants were instructed to shift their gazes between marks on a slated plane (which varied simultaneously in both depth and horizontal distance, so that gaze shifts between the marks required both version and vergence movements). The researchers reasoned that the needle-threading task did not result in a comparable coordination of microsaccadic movements due to having relatively impoverished 3D depth cues (and thus providing a weak binocular disparity signal). However, it is possible that vergence movements, version movements, or their coordination, were not critical for the needle-threading task.

The combined findings from the above studies indicate that microsaccades enhance performance in certain high-acuity tasks by moving gaze very precisely to key locations, at the right times. 

Further research is needed to understand the contributions of FEMs to aiming and shooting. Whereas microsaccades could be transiently suppressed just the trigger is pulled, possibly aiding the shooter’s aim, this possibility has not been tested with actual aiming and shooting tasks, where sports-specific demands can differ from those in other high-acuity tasks. For example, elite shooters not only have extensive practice in precisely aligning their aim to small targets, but they also may need to track targets moving in unpredictable directions from unpredictable starting locations (as in clay shooting).

One study explored visuo-motor differences between high-level clay target shooters and inexperienced controls in a visually guided saccade task: participants were instructed to quickly saccade only towards red targets (at 6° eccentricity), and to maintain fixation if green distractors appeared instead (at 1.5° or 3° of eccentricity) (81). Expert shooters produced large saccades (6°) with faster latencies than novices. After training for ten 30-minute sessions on the saccade task, one of the study’s authors also made saccades with equivalent latencies to those of expert shooters. It is unclear if such speeded latencies might extend to microsaccadic eye movements produced during aiming. 

This same study also assessed the fixational stability of expert and novice shooters, in terms of their gaze deviation from the fixation point. Whereas the fixation patterns of the two groups were comparable in the presence of shooting targets, novice shooters had more unstable fixations than experts in the presence of distractors. Future research might assess the relative contributions of microsaccades and drift (as opposed to larger saccades) to the gaze (in)stability of experts and novices. Ideally, this work should be conducted in real-world scenarios and involve elite shooters, given that expertise in shooting tasks may not result in oculomotor differences in contrived experimental settings. 

### Unwanted FEMs in applied scenarios

FEMs, by definition, impede precise fixation. The resulting retinal motion can create difficulties in certain applied and clinical domains. For example, some neuro-ophthalmic examinations (such as pupillographic campimetry) require the patients’ accurate fixation during testing, which can be challenging—particularly in the presence of pathological FEMs. Thus, accounting for FEMs is necessary for the meaningful evaluation of visual field defects, as well as for tracking their progression (82). Importantly, many patients with neurological or ophthalmic conditions have FEMs of increased amplitudes and/or rates (11, 83), which can make clinical examinations more difficult than in patients that do not suffer from fixational impairments. 

**Figure 3.**
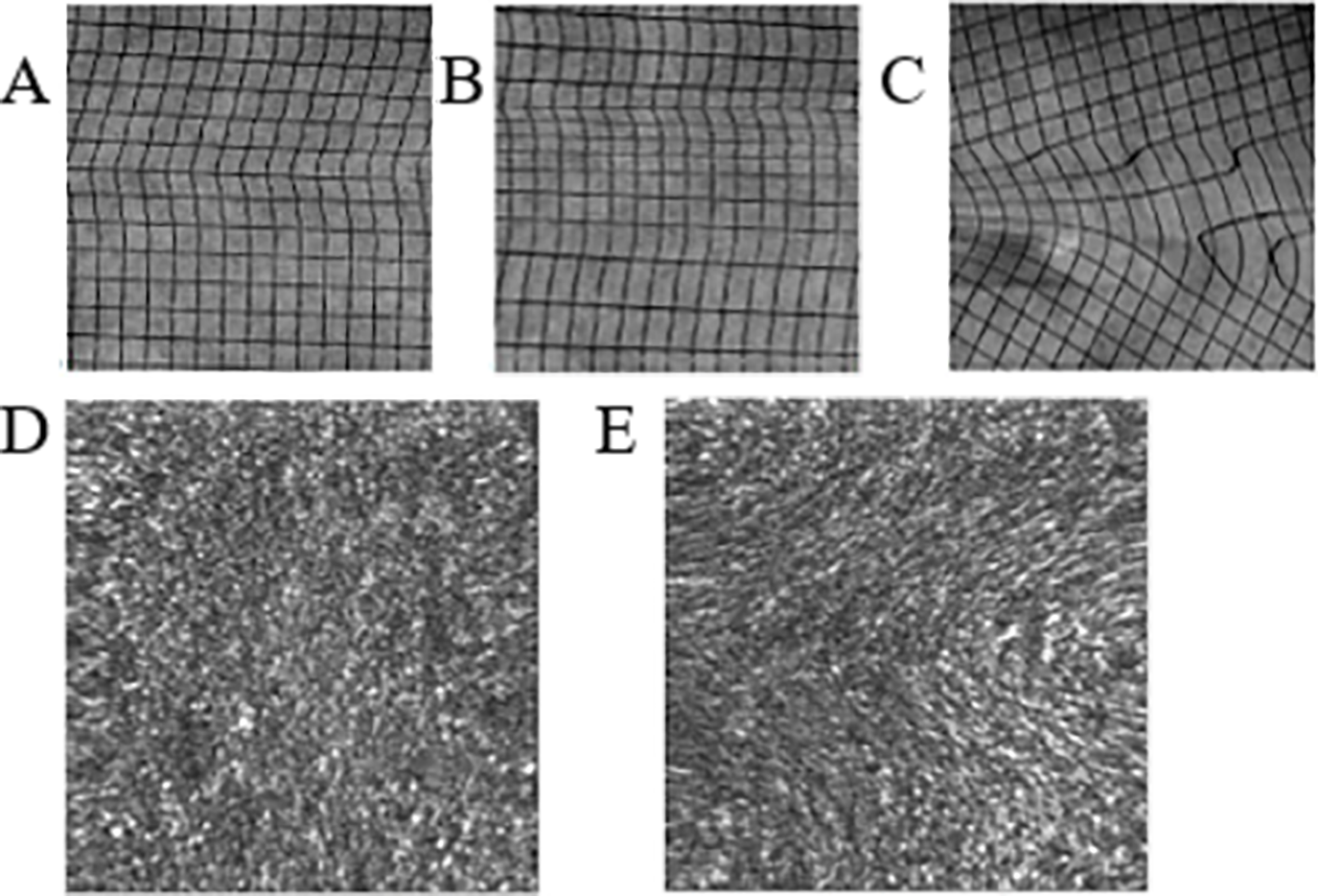
A-C) Distortions in scanned images, produced by (A) horizontal, (B) vertical, and (C) torsional motion within a single scan. (D-E) Successive frames from a 1° square region of foveal human retina. Panel D is typical, but a rapid horizontal eye movement has distorted the image in panel E. From Stevenson and Roorda (85).

FEMs can also hamper the creation of medical images of a patient’s retina, such as with scanning light ophthalmoscopy (with or without adaptive optics), by generating image warping artifacts and the ensuing image processing difficulties (84, 85)—see **Figure 3**.

Post-processing removal of retinal motion relies on an important prerequisite: having accurate FEM measurements to begin with. Attempts to solve these problems have resulted in an innovative means of recording FEMs with arcsecond resolution, via adaptive optics. 

Adaptive optics scanning laser ophthalmoscopes can provide measures of retinal position at the level of individual cones, and record the movement of these cells during microsaccades, drift and tremor (86, 87). In fact, adaptive optics systems are *only* able to effectively measure small eye movements (rather than large saccades), because their fields of view are very limited in size (8° or smaller). 

FEMs also produce “fixation jitter” in gaze-assisted computer cursors, making eye movement cursors less precise than standard mouse cursors (88). Except for this limitation, eye movements might serve as a reliable cursor, enhancing the accessibility and usability of smartphones, projected displays, and other devices (as well as serve as accurate indicators of users’ attentional allocation). An improved understanding of FEMs may lead to methods that overcome these obstacles. Indeed, some gaze-controlled cursors already consider microsaccades and drift in their implementation. For example, Miniotas and Špakov (89) devised an algorithm aimed at disregarding the shifts in gaze position due to FEMs on gaze cursors. Other designs address FEM-induced gaze position shifts by making buttons and menus large enough that small eye movements do not take the user’s gaze away from intended targets (90). A main disadvantage of the latter solution is that larger design features take up screen space that could otherwise be used for additional control and display elements. 

In situations where tasks are impossible or difficult for humans to complete, robots and other devices are increasingly controlled via teleoperation. Gaze-based commands can complement systems relying on visually evoked potentials to operate wheelchairs, robots, or drones (91, 92). However, involuntary microsaccades may unintentionally signal functions to activate—the so-called “Midas touch” problem (93). Using gaze as a means of issuing commands might improve telemedicine and other remote applications once the above-discussed difficulties concerning gaze-assisted cursors are solved.

Inadvertent microsaccades can also introduce artifacts in EEG recordings: these include small corneo-retinal artifacts (94, 95), as well as microsaccadic modulation of evoked potentials, which can mimic changes in induced gamma band power (96). Neuroimaging studies have started to use high-resolution eye tracking to remove trials with microsaccades from analysis (97). 

### Limitations intrinsic to FEM measures

While the study of FEMs promises fruitful applications to everyday life situations, some intrinsic limitations exist due to the nature of FEM themselves. For example, conditions involving excessive blinking would limit the usefulness of all eye movement measures, including FEMs. 

The link between microsaccades and the state of the observer (covert attention, etc.) has been consistently observed, but it does not appear to produce perfect correspondence, as microsaccade production may jointly depend on low-level visual stimulation features (26, 27, 49, 98), oculomotor correction (99, 100, 101), and/or the state of the observer. Even in task contexts where tight links between microsaccades and attention are expected, more than 10% of microsaccades do not share the same direction as spatial attention (7). Microsaccadic attentional indices therefore necessarily have some level of noise and do not serve as perfectly accurate measures of attentional focus. This limitation could be exacerbated in applied contexts with functional limits as to the accuracy and timing of assessments.

Though various microsaccadic features have been linked to task performance and fatigue in a variety of tasks—including during fixation, guided viewing and visual search (6, 28, 65), some applied tasks may require frequent large saccades, thereby reducing microsaccade occurrence by virtue of the common oculomotor generator underlying microsaccade and saccade production (27, 49, 101, 102, 103). The fact that microsaccades are transient events could also limit their potential for indexing an individual’s state in some contexts. Drift and tremor could provide more continuous measures, but few studies have focused on drift (and none on tremor) in applied contexts, due to the difficulties inherent to its measurement.

### Potential future real-world applications

FEM research has often been limited to artificial contexts, largely due to equipment and methodological limitations: Most head-or desk-mounted high-resolution and high-frequency eye trackers cannot follow gaze while a participant is walking, running, or engaged in many daily activities. However, improvements in commercially equipment, and the increasing affordability of high-speed eye trackers, have expanded the range of feasible scenarios for eye-tracking data collection. Some innovative choices in experimental paradigms and camera setups have further allowed for increasingly realistic experimental contexts. 

Accordingly, microsaccadic features (among a range of FEM dynamics) are being considered in many research scenarios, in and out of the lab, where they were not traditionally studied. Below, we discuss further future possibilities.

#### Image processing

A common challenge in image processing is the difficulty in identifying task-relevant image regions. Microsaccade dynamics have been linked to image informativeness, which combines bottom-up and top-down factors (26). Thus, microsaccadic features may prove fruitful in image processing applications, including semantics-aware image segmentation, photo cropping, object/face recognition, and image post-processing (e.g. selectively adjusting the tone or style of semantically-relevant image regions). Some researchers have proposed that microsaccades recorded noninvasively with glasses-based eye trackers could reveal what kind of information a user is interested in, and then use the data to display related information (104). 

#### User experience and marketing

Large saccades have been applied to improve customers’ ‘quality of experience’ with product/services, in contexts ranging from predicting intent to playing a movie (so that the delay before the video begins can be shortened, (105)) to lowering the bandwidth of the visual periphery in virtual reality and video displays to achieve higher resolution in foveal viewing regions (e.g. 106). Future work might explore similar uses for microsaccades and/or enable microsaccade-specific manipulations (for instance, scaling the quality of video displays based not only on current gaze position, but also on covertly attended regions indicated by biases in microsaccade directions). That is, because microsaccade directions can point to the location of covert spatial attention (7, 8), future neuromarketing studies might use microsaccade direction biases to gauge the location and strength of covert customer interest on services and products (107).

Microsaccade measurements may be likewise useful in educational settings, by assessing the user experience of students. FEM recordings could help identify what elements of an e-learning platform best capture student interest, as well as help detect when students become fatigued due to excessive cognitive load and/or time-on-task. 

Additionally, improved models of microsaccadic biomechanics may be used to help prevent visual fatigue in a variety of displays, including virtual environments (108).

#### Healthcare

FEMs are altered in a wide variety of ophthalmic and neurologic conditions (11). However, FEM assessments are not typically used for diagnostic purposes, or to fine-tune and track the efficacy of treatments over time (for example, increasing or decreasing a medication dose when FEM dynamics are altered beyond specified parameters). Yet, there are indications that a better understanding of these eye movements could improve patient health and safety. For example, the production of FEMs by ophthalmic patients during femtosecond laser-assisted cataract surgery has been linked to certain post-surgical complications, such as increases in the rate of anterior capsule tears (109). 

The development of low-cost, noninvasive eye movement-based screening tools for medical conditions could have significant real-world impact. For instance, the inclusion of FEM measurements in screening methods could expand and improve the early identification of disorders, leading to timely diagnoses and treatments. Modelling FEMs in virtual or augmented environments may moreover provide insights into how fixation dynamics change in diseased or injured states (108).

Mental health counselling may also benefit from FEM measurements. FEM dynamics have been linked to physiological states such as arousal (6, 28, 65, 66). Establishing a comparable link to psychological states could help mental health practitioners track a client’s status or the success of treatment plans.

Tracking large and small eye movements may also help assess fatigue in healthcare workers and provide insight into fatigue-related errors (65, 110). Fatigue and burnout in healthcare workers results from high workloads, long work cycles, shift work, and sleep loss, among other factors (111, 112, 113). Mental fatigue is widely cited as a contributing factor for catastrophic errors (114). Fatigue can have direct adverse impacts on healthcare personnel, such as job dissatisfaction, increased risk of burnout, and increased rates of on-the-job injuries. Fatigue in healthcare workers can also create a host of secondary effects on patients, given that fatigued workers suffer diminished reaction times, impaired communication, inattentiveness, and lapses in memory. Sleep deprivation and fatigue can therefore lead to medical errors (115). For example, fatigue can deteriorate the ability of radiologists to detect nodules, fractures, or other abnormalities in medical images (110, 116). Fatigued paramedics may also have an increased error rate, as well as an increased rate of accidents due to impaired driving ability (116).

In the COVID-19 pandemic, the healthcare system has shifted towards virtual care (117) and some of the regulations and systems that were quickly created to allow this shift could stay in place after the pandemic subsides—or similar transformations may occur during future pandemics. Eye tracking tools for monitoring fatigue might be integrated into telemedicine workflows, where physicians and other healthcare personnel already use cameras to consult with patients (which might be accessorized to facilitate eye tracking). FEM assessments added to telehealth stations might also aid diagnosis and the tracking of treatment efficacy. 

Fatigue assessments in healthcare workers may be a more pressing issue for in-person consultations, however, in that worker fatigue could increase the risk of infection from emerging diseases (118). Extensive use of personal protective equipment and the need to take on additional tasks can further fatigue healthcare personnel during healthcare crises that overwhelm the medical system. Some work shifts have been altered to comply with social distancing guidelines (i.e. decreasing the overlap between different shifts of healthcare personnel), rather than to minimize fatigue or optimize performance. Robust measurements of fatigue—including those provided by FEM measurements—may clarify the physiological and psychological outcomes for healthcare employees working in such extraordinary circumstances and help identify potential interventions.

#### Deception

People generally produce FEMs unintentionally and are unaware of their occurrence (20). It follows that FEM measures may provide a non-intrusive and surreptitious assessment of mental states that participants cannot easily access or manipulate. Thus, FEM parameters could indicate arousal levels or attentional foci that an individual tries to conceal, thereby serving to reveal (intended) deception. If proven effective, such measures could provide tools for national defense, counter-terrorism, and law enforcement fields, as well as for psychiatric and medical evaluations, as suggested by (107). 

#### Visual prosthetics

Eye movements, including microsaccades, are critical to designing visual prosthetics that faithfully reproduce natural vision (119, 120, 121). The intended recipients of visual prostheses are typically expected to have similar numbers of (micro)saccades as normally-sighted persons (122, 123). These eye movements can be directly accounted for by implants relying on intraocular light-sensing circuitry (such as in the Alpha IMS subretinal implant). Because the photovoltaic conversion happens within the eye, eye movements alter both the light entering the eye and the corresponding electrical stimulation from the implant (124, 125). This process accounts both for large eye movements and for FEMs (126, 127). However, visual prosthetics that rely on external cameras need to account for eye movements to provide accurate visual percepts to the implanted patient. A prosthesis could account for the perceptual effects of oculomotor movements by tracking eye movements and then adjusting the output in real-time (120). Because FEMs affect perception, these cameras will need to reliably and accurately detect FEMs before natural vision can be fully reproduced. FEM measures may also help evaluate vision restoration by an implant, by comparing the oculomotor behavior of implanted patients to that of controls (11, 123). 

#### Expertise and worker performance

An important path for future FEM applications will be to gain further understanding of gaze parameters in professional environments and task scenarios where FEMs are already known to be relevant. For example, though microsaccades are known to be affected by fatigue—as discussed earlier (28, 31, 34)—researchers have yet to determine specific cutoffs that may be used as fitness-for-duty evaluation criteria for flight crews, drivers at increased risk for motor vehicle collisions, and other critical personnel. Thus, a more precise understanding of FEM parameters could be used to develop real-time monitoring devices for fatigue and other operator states that lead to critical task failures. 

FEMs may also be used to harness the professional knowledge of workers engaged in specialized visual tasks, particularly those performed in restricted visual areas (such as small screens or panels) where larger eye movements are less relevant. In addition, FEMs might help ascertain differences in expertise among operators. For example, because microsaccade dynamics change with cognitive workload (6), microsaccadic features could help assess the success of training regimes that lessen the cognitive load of operators in a variety of contexts. FEM differences between expert and novice operators, if found, might be used to improve human-computer interfaces, image classifiers, or training protocols.

## Conclusions

We reviewd the literature on real-world and ecologically-valid applications of FEMs, with a focus on microsaccades. Analysis of FEMs has revealed relationships between these movements and fatigue, hypoxia, and vestibular sensory deprivation, as well as other changes in health status. FEM measurements also provide insights into the allocation of covert attention, and what information is important during a given task. Measuring FEMs in ecologically-valid contexts has moreover improved our understanding of the role that these motions play in visuomotor performance and perceptual judgments. Attempts to counteract unwanted FEMs have additionally led to certain technological innovations, such as the creation of novel eye-tracking systems that allow the removal of FEM-based retinal motion artifacts from medical images. Future research may explore not only how FEMs affect performance in applied contexts, but also how FEM measurements can be harnessed to improve execution and/or reduce work hazards in a variety of real-world scenarios—especially as eye tracking technologies continue to advance. 

## Ethics and Conflict of Interest

The author(s) declare(s) that the contents of the article are in agreement with the ethics described in http://biblio.unibe.ch/portale/elibrary/BOP/jemr/ethics.html and that there is no conflict of interest regarding the publication of this paper. 

## Acknowledgements

This work was supported by the Empire Innovator Program, State of New York and the National Science Foundation (Award 1734887 to SMC and SLM; Award 1523614 to SLM). We thank Dr. Katherine Alexander for feedback during the preparation of this manuscript.
